# Towards cross-center head and neck cancer detection: a multi-level domain alignment exploration

**DOI:** 10.3389/frai.2026.1882547

**Published:** 2026-08-06

**Authors:** Jiaqi Zhao, Yongjie Liang, Ziwei Zhu, Yu Wang, Cheng Lu, Xiaohui Duan, Yuexiang Li

**Affiliations:** 1 School of Life Sciences and Medical Engineering, Guangxi Medical University, Nanning, China; 2 Center for Genomic and Personalized Medicine, Guangxi Key Laboratory for Genomic and Personalized Medicine, Guangxi Collaborative Innovation Center for Genomic and Personalized Medicine, Guangxi Medical University, Nanning, China; 3 Guangdong Cardiovascular Institute, Guangdong Provincial People's Hospital (Guangdong Academy of Sciences), Southern Medical University, Guangzhou, China; 4 Department of Radiology, Sun Yat-sen Memorial Hospital, Sun Yat-sen University, Guangzhou, China; 5 Guangdong Provincial Key Laboratory of Malignant Tumor Epigenetics and Gene Regulation, Medical Research Center, Sun Yat-sen Memorial Hospital, Sun Yat-sen University, Guangzhou, China; 6 Department of Radiology, Guangdong Provincial People's Hospital (Guangdong Academy of Medical Sciences), Southern Medical University, Guangzhou, China; 7 Guangdong Provincial Key Laboratory of Artificial Intelligence in Medical Image Analysis and Application, Guangzhou, China; 8 Medical Research Institute, Guangdong Provincial People's Hospital (Guangdong Academy of Medical Sciences), Southern Medical University, Guangzhou, China; 9 Nanning Research Institute, Guilin University of Electronic Technology, Nanning, China; 10 Guangxi Academy of Artificial Intelligence, Nanning, China

**Keywords:** 3D CT classification, cross-center deployment, domain generalization, head and neck cancer, medical image analysis

## Abstract

**Introduction:**

Deep learning models for head and neck cancer (HNC) detection from computed tomography (CT) hold significant promise for improving early detection—a critical priority given that 5-year survival drops from 84% for localized disease to 39% for metastatic cases. However, robust cross-center deployment remains challenging because scanner vendors, acquisition protocols, reconstruction Q15 kernels, and patient populations vary across hospitals. To address this challenge, we propose MDA-Net, a multi-level domain generalization framework for cross-center 3D CT classification. The final MDA-Net configuration is deliberately designed as a training-time generalization strategy: it does not use target-domain batches, target-domain labels, target-domain statistics, or adaptive updates during inference. Instead, it performs a single forward pass with fixed parameters on each unseen target CT volume.

**Methods:**

Our framework integrates three complementary levels: (i) input-level inverse frequency weighted sampling, Fourier Domain Adaptation (FDA), and Mixup augmentation to mitigate class imbalance and diversify source styles; (ii) feature-level IBN-enhanced 3D ResNet-18, DANN, and CORAL to learn style-invariant and statistically aligned representations; and (iii) optimization-level Sharpness-Aware Minimization (SAM) and Stochastic Weight Averaging (SWA) to stabilize training under style perturbation. We evaluate MDA-Net on a multi-center dataset comprising 1,081 cases from three hospitals under a strict leave-one-center-out protocol.

**Results:**

On the primary Foshan target domain, MDA-Net improves AUC from 0.5121 to 0.5869 and ACC from 0.4829 to 0.6000; reverse validation on another held-out center further yields AUC = 0.5887. Our stepwise ablation demonstrates that IBN provides the largest single-component gain, while FDA and CORAL become beneficial only when combined with SAM, SWA, Mixup, and a moderate adversarial weight of λ = 0.1.

**Conclusions:**

These results position MDA-Net as an exploratory yet practical, privacy-preserving, and computationally efficient domain generalization strategy for cross-center HNC detection.

## 1 Introduction

Head and neck cancer (HNC) represents one of the most prevalent and lethal malignancies worldwide. According to GLOBOCAN 2022 statistics, over 930,000 new cases and approximately 460,000 deaths occur annually (Sung et al., 2021), with oral and laryngeal cancers constituting the majority of this disease burden. HNC encompasses multiple anatomically complex sites—including the oral cavity, oropharynx, hypopharynx, larynx, sinuses, nasal cavity, and salivary glands—exhibiting significant heterogeneity. Despite advances in radiological techniques, minimally invasive surgery, and precise radiotherapy, HNC continues to be characterized by high local recurrence rates (30%–50%), strong invasiveness, and severe functional impairment affecting swallowing, speech, and respiration. The 5-year survival rate remains approximately 50%–60% overall, dropping below 30% for advanced cases (Johnson et al., 2020). Notably, SEER database statistics reveal a dramatic survival gap: 84% for localized stage versus 39% for metastatic disease, underscoring the decisive importance of early detection and accurate staging (Siegel et al., 2024; Davaatsend et al., 2023; National Cancer Institute, 2024; Saba and Posner, 2025).

In clinical practice, computed tomography (CT) imaging serves as an indispensable tool for HNC assessment, offering unique advantages in visualizing bone cortex destruction, calcification, and acute hemorrhage. Multi-phase enhanced CT clearly delineates spatial relationships between tumors and critical structures such as the carotid sheath and skull base (Liao et al., 2012). Compared to physical examination or serological markers, CT provides high-resolution three-dimensional anatomical information, objectively displaying tumor infiltration patterns, lymph node metastasis, and vascular encasement. With fast scanning (< 5 minutes), high spatial resolution (0.5 mm slice thickness), widespread availability, and relatively low cost, CT has become fundamental for preliminary assessment, radiotherapy planning, and follow-up management (Gillies et al., 2016). Given this clinical reliance on CT, deep learning-based automated classification systems hold significant promise to improve diagnostic efficiency, reduce subjective interpretation variability, and extract quantitative biological information invisible to the naked eye through radiomics, thereby providing objective evidence for clinical decision-making (Chartrand et al., 2017; Chen et al., 2021; Hosny et al., 2018).

However, the development of such robust models faces a critical “data bottleneck” that directly hinders their real-world deployment. The intricate anatomy of the head and neck region, combined with invasive tumor growth patterns and indistinct boundaries, makes image annotation heavily dependent on experienced specialists with 5–10 years of expertise. Manual lesion delineation and classification require substantial time (30–60 minutes per case) and incur high costs. More importantly, even when annotated data are available, significant variations exist across institutions in scanning protocols, equipment manufacturers, reconstruction algorithms, and interpretation styles. These discrepancies severely limit cross-institutional generalization, causing substantial performance fluctuations when models encounter different equipment parameters or hospital settings—a phenomenon known as “domain shift” or “dataset drift” (Guan and Liu, 2021; Zhou et al., 2022). Consequently, while convolutional neural networks (CNNs) and vision Transformers achieve >90% accuracy on single-institution datasets, cross-institution deployment often results in 20%–40% performance degradation. This has led to a gap between research performance and practical application, which is a key obstacle for the clinical application of artificial intelligence in the field of head and neck tumors.

Given these challenges, we focus on a clinically pragmatic task: binary classification for HNC detection from CT scans. This task—distinguishing between malignant and benign lesions, or between lesion and non-lesion—directly addresses the early detection imperative highlighted by the SEER survival gap. Within clinical workflows, such binary decisions serve as efficient screening filters, enabling rapid triage of suspicious cases for specialist review. They also provide foundational inputs for downstream tasks including tumor segmentation, staging, and treatment response assessment (Illimoottil and Ginat, 2023). Importantly, compared to pixel-level segmentation or multi-class subtyping, binary classification requires substantially less annotation effort and computational resources, making it particularly viable for deployment in resource-constrained clinical settings.

To overcome the generalization barrier that undermines these clinical applications, domain generalization (DG) has emerged as a promising paradigm. DG aims to train models using only labeled source-domain data to extract domain-invariant features, enabling stable performance in unseen target domains without requiring target labels or adaptive target-domain statistics—an essential property for privacy-preserving clinical scenarios (Wang et al., 2022). Applying DG to HNC CT classification poses unique challenges: the anatomical complexity of the region demands fine-grained feature extraction, while domain shifts across centers can manifest both as global style variations (e.g., scanner characteristics) and local texture discrepancies (e.g., reconstruction kernels). These dual challenges call for a comprehensive alignment strategy that operates across multiple levels of the learning pipeline. For clinical translation, such a strategy should satisfy three practical constraints: (i) no adaptive use of target-domain cohorts, preserving patient privacy; (ii) no target-domain annotations, reducing deployment cost; and (iii) single-pass inference, enabling efficient use in resource-limited clinical settings.

To address this, we propose MDA-Net, a unified domain generalization framework, aiming to enhance the robustness to unseen target domains. Our MDA-Net uses 3D ResNet-18 as the backbone network, while integrating complementary alignment strategies across three levels: inverse frequency weighted sampling at the input layer, FDA and Mixup to alleviate imbalance and diversify the source domain style; adversarial domain alignment (IBN and CORAL) at the feature layer to learn style-invariant and statistically aligned representations; and robust training at the optimization layer (SAM and SWA) to flatten the loss terrain. During testing, the learned encoders and classifiers are frozen, and each target CT volume is processed through a single forward pass. This design of MDA-Net directly addresses the practical gap between source domain training and inference on unseen centers, while maintaining the privacy protection and computational efficiency of the method. Our contributions are in four aspects:

- **Three-level domain generalization framework**. We proposed MDA-Net, which is a framework that collaboratively combines input layer style diversity, feature layer adversarialness and statistical alignment, as well as optimization layer robust training, for cross-center HNC detection.
- **IBN-centered style-invariant representation learning**. We integrate IBN into the early layers of the 3D ResNet-18 encoder and couple it with DANN and CORAL, enabling the model to suppress center-specific style variation while preserving task-discriminative anatomical features.
- **Efficient and privacy-preserving inference**. We have demonstrated that the final MDA-Net achieves its gain without performing adaptive target domain updates. Moreover, the trained model uses frozen parameters for a single forward pass, which to some extent reduces the inference cost and avoids the use of target domain queues or annotations.
- **Systematic evaluation and practical guidelines**. To more clearly demonstrate our progress, we conducted a progressive ablation study, a sampling strategy isolation experiment, and an adversarial weight scan, thereby demonstrating that IBN is the core contributor, while FDA/CORAL requires SAM/SWA stabilization and moderate adversarial weights to achieve effective collaboration.

## 2 Related works

### 2.1 Medical image classification for head and neck cancer

Medical image classification typically follows the deep supervised learning paradigm, where labeled data pairs (*x, y*) are used to train neural networks *f*_θ_ to learn the mapping from input images to disease category probabilities *p*(*y*∣*x*). In the context of head and neck cancer (HNC) detection from CT, architectural choices, training strategies, and clinical usability considerations collectively shape model development.

#### 2.1.1 Architectural backbones

Pre-trained residual networks (ResNet) (He et al., 2016) and densely connected networks (DenseNet) (Huang et al., 2017), originally developed for natural image domains, are widely adopted as feature extraction backbones. Their cross-layer connection mechanisms effectively alleviate gradient vanishing and facilitate the capture of multi-scale morphological features in HNC lesions. More recently, ConvNeXt (Liu et al., 2022) has modernized pure convolutional architectures by incorporating Transformer design principles, achieving improved representational capacity while maintaining computational efficiency. Concurrently, state space models (SSM)—exemplified by Mamba (Gu and Dao, 2024) and VMamba (Liu et al., 2024)—have emerged as promising alternatives, offering global receptive fields with linear computational complexity, a property particularly advantageous for high-resolution medical image analysis.

While these architectures focus on backbone design, recent advances in medical foundation models have explored vision-language pre-training on large-scale CT data. For instance, Merlin (Blankemeier et al., 2026) learns from abdominal CT scans, electronic health records, and radiology reports, demonstrating strong generalization across 44,098 CT scans from multiple external sites.

Similarly, CT-CLIP and CT-CHAT (Hamamci et al., 2026) leverage the public CT-RATE dataset of 25,692 3D chest CT scans with paired radiology reports, enabling generalist vision-language understanding of volumetric medical images.

#### 2.1.2 Training strategies for imbalanced data

Class imbalance is a persistent challenge in HNC datasets, where early-stage samples are often scarce. Beyond standard cross-entropy loss, Focal Loss (Lin et al., 2017) mitigates this by down-weighting easy-to-classify examples, thereby focusing model attention on challenging cases. Ranking-based loss functions, such as AUC-maximization and Rank loss (Yao et al., 2016), directly optimize classifier ranking ability, improving sensitivity to positive cases. For data augmentation, Mixup (Zhang et al., 2017) and CutMix (Yun et al., 2019) enhance generalization by minimizing neighborhood risk, while automated augmentation strategies like RandAugment (Cubuk et al., 2020) effectively expand the diversity of limited labeled data.

#### 2.1.3 3D volume processing

HNC CT scans are inherently volumetric, necessitating specialized processing strategies. Existing research primarily follows three technical routes: (i) direct volumetric feature learning using 3D convolutional networks (e.g., 3D ResNet or nnU-Net) (Isensee et al., 2021); (ii) conversion of 3D data into representative 2D slices (axial, coronal, and sagittal) with final predictions aggregated via voting or feature fusion (Cai et al., 2020); and (iii) 2.5D approaches that process multi-planar reconstructions or adjacent slice stacks to balance spatial context and computational efficiency.

#### 2.1.4 Transfer learning and explainability

Transfer learning is particularly valuable in medical small-sample scenarios. Pre-training on ImageNet (Russakovsky et al., 2015) or large-scale medical image datasets (e.g., REMEDIS) (Azizi et al., 2021), followed by fine-tuning on target domain data, significantly accelerates convergence and improves performance. Beyond outputting class probabilities, modern medical classification systems increasingly emphasize clinical usability through explainability. Gradient-based class activation mapping (Grad-CAM) (Selvaraju et al., 2017) and its variants highlight decision-critical regions, enabling radiologists to verify the clinical plausibility of model focus areas and thereby enhancing trust in human-machine collaborative diagnosis.

### 2.2 Domain generalization for medical imaging

Due to differences in scanners, protocols, and patient populations, this leads to domain shift, which poses significant obstacles for deploying deep learning models across multiple clinical centers. Domain generalization (DG) enables models to maintain robust performance on unseen target domains by training them on labeled source domains without accessing data from the target domain. (Yang et al., 2026) conducted a comprehensive review of unsupervised domain adaptation in medical imaging, categorizing the methods as adversarial-based, reconstruction-based, and self-supervised. In this paper, based on our proposed MDA-Net framework, we divide the methods into three levels according to their functional hierarchy: input layer, feature layer, and optimization layer.

For a comprehensive overview of unsupervised domain adaptation in medical imaging (Yang et al., 2026), which systematically categorizes existing UDA techniques and summarizes commonly used public benchmarks.

In the context of semi-supervised segmentation (Zhou et al., 2025), which employs dual-score adaptive filtering and elliptical constraint pseudo-label refinement techniques to improve the segmentation effect of the fetal head in ultrasound images.For semi-supervised tumor segmentation in CT scans (Jin et al., 2025), which iteratively refines pseudo-labels and applies adaptive copy-paste supervision to improve segmentation performance.

#### 2.2.1 Input-level strategies

To mitigate appearance variations caused by differences in equipment manufacturers and scanning protocols, intensity normalization techniques—such as Hounsfield unit truncation and window width/level standardization—are widely employed. Style transfer methods (Huang and Belongie, 2017) further reduce domain gaps by synthesizing images with diverse stylistic attributes. MixStyle (Wang et al., 2021) extends this concept by mixing feature statistics across domains in the feature space, effectively augmenting the diversity of domain-specific characteristics during training. Additionally, self-supervised contrastive learning approaches, including SimCLR (Chen et al., 2020) and MoCo (He et al., 2020), leverage large-scale unlabeled medical images for pre-training, yielding more generalizable low-level representations that are less sensitive to domain variations. These input-level techniques directly manipulate data or its representations to enhance robustness against domain shifts.

#### 2.2.2 Feature-level strategies

A major branch of DG methods applies explicit distribution alignment constraints in feature space to learn domain-invariant discriminative features. Representative approaches include Maximum Mean Discrepancy (MMD) (Gretton et al., 2012), which minimizes the distance between domain distributions in a reproducing kernel Hilbert space; Second-Order Statistical Alignment (CORAL) (Sun and Saenko, 2016), which aligns domain covariances; and Domain-Adversarial Neural Networks (DANN) (Ganin et al., 2016), which promotes domain invariance through an adversarial domain classifier. From an architectural perspective, Domain-Specific Batch Normalization (DSBN) (Chang et al., 2019) maintains separate normalization statistics for different source domains, while hybrid normalization designs such as IBN combine instance- and batch-level statistics to suppress style variation while retaining discriminative content. Domain decoupling methods (Peng et al., 2019) further extend this idea by explicitly separating domain-specific and domain-invariant feature components through dedicated encoding pathways. These feature-level techniques aim to learn representations that are invariant to domain variations.

#### 2.2.3 Optimization-level strategies

To improve robustness against distribution shifts, Group Distributionally Robust Optimization (GroupDRO) (Sagawa et al., 2019) optimizes performance on the worst-performing source domain, ensuring equitable generalization across all observed domains. Shi et al. (2022) explicitly optimizes domain inter-gradient alignment through meta-learning, simulating domain shifts by partitioning source domains into meta-training and meta-testing sets. Invariant Risk Minimization (IRM) (Ahuja et al., 2020) seeks to learn causal mechanisms that remain invariant across source domains, avoiding over-reliance on spurious correlations that may not hold in unseen domains. Meta-learning-based DG methods, particularly MAML variants (Finn et al., 2017), simulate domain shifts during training by partitioning source domains into meta-training and meta-testing sets, forcing models to acquire meta-knowledge that enables rapid adaptation to new domains (Li et al., 2018). Contrastive learning has also been integrated into DG frameworks, constructing domain-invariant embedding spaces by pulling similar cross-domain samples closer while pushing dissimilar ones apart (Kim et al., 2021). These optimization-level strategies modify the training objective or procedure to enhance cross-domain generalization.

In summary, existing DG methods for medical imaging span a broad spectrum—from input-level style normalization and feature-level distribution alignment to optimization-level robust training. However, many approaches focus on only one or two levels of alignment, potentially leaving vulnerabilities to domain shifts at other levels. In contrast, MDA-Net integrates complementary strategies across three levels within a unified framework, achieving robust cross-center HNC detection while preserving privacy and inference efficiency.

## 3 Methodology

### 3.1 Problem formulation

We consider the binary classification task of head and neck cancer (HNC) detection from 3D CT scans under a multi-source domain generalization setting. The training set consists of *K* source domains, as formulated in Equation 1:


$$\begin{array}{l} D_{s}=\overset{K}{\underset{k=1}{⋃}}D_{k},\;D_{k}={\left\{\left(x_{i}^{\left(k\right)},y_{i}^{\left(k\right)}\right)\right\}}_{i=1}^{N_{k}}, \end{array}$$ (1)

where $x_{i}^{\left(k\right)}\in {\mathbb{R}}^{C\times D\times H\times W}$ denotes the 3D CT volume (with channel *C*, depth *D*, height *H*, and width *W*), and $y_{i}^{\left(k\right)}\in \left\{0,1\right\}$ is the binary label indicating the presence of HNC. The domain label $d_{i}^{\left(k\right)}=k$ indicates the source hospital or center, which is available during training but not used during inference on unseen target domains.

Our goal is to learn a model f:X→Y using only labeled source domain data $D_{s}$ that generalizes well to an unseen target domain $D_{t}={\left\{x_{j}\right\}}_{j=1}^{N_{t}}$ without requiring any target-domain data during either training or inference. This setting is particularly relevant for privacy-sensitive clinical scenarios where target-domain data cannot be shared or annotated.

### 3.2 Overview of MDA-Net

**Figure 1:**
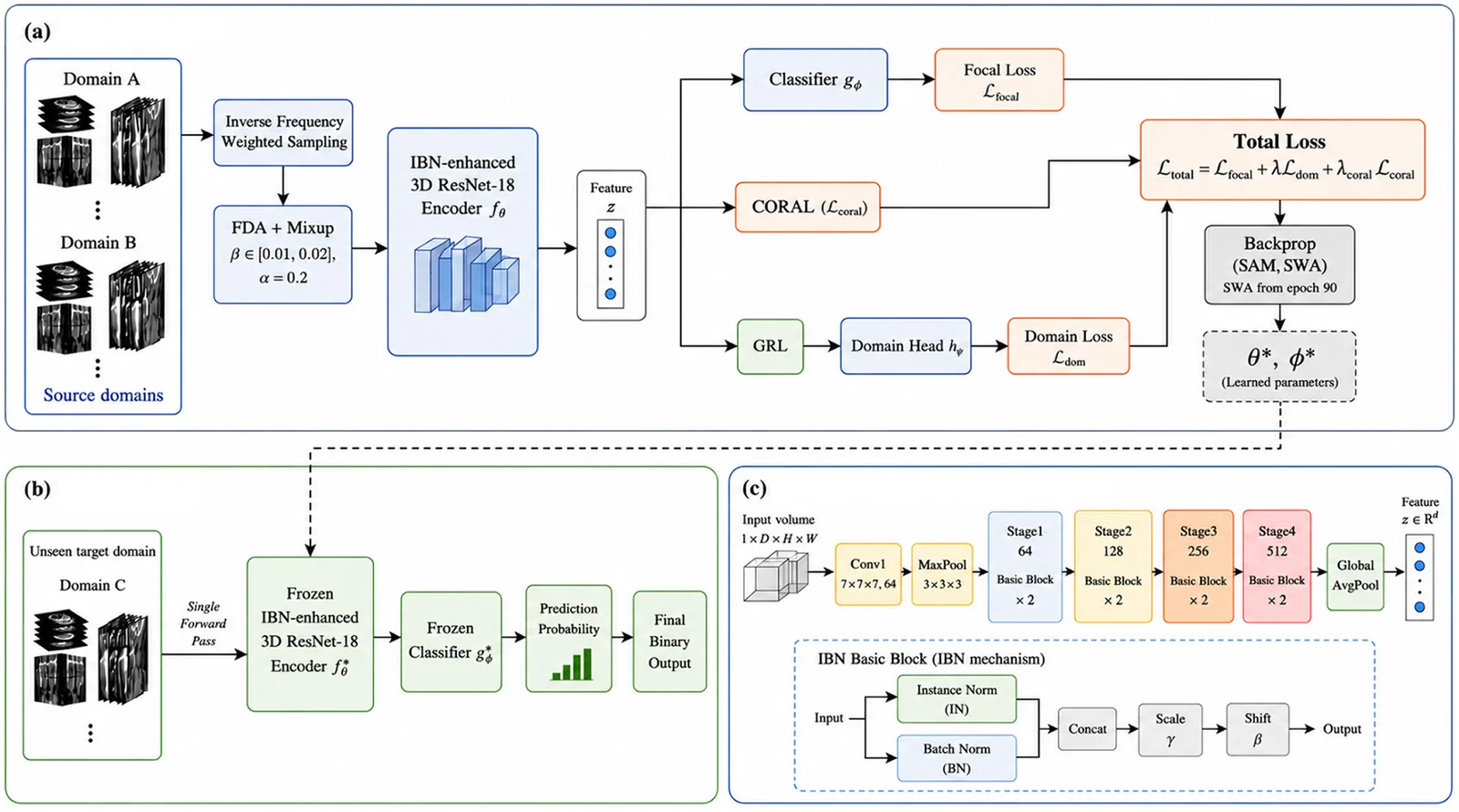
Overview of MDA-Net (training-time only domain generalization). **(a)** Training phase: 3D CT volumes from multiple source domains (Domain A, B, ...) first undergo inverse frequency weighted sampling, followed by input-level style perturbation via FDA (β ∈ [0.01, 0.02]) and Mixup augmentation (α = 0.2). The IBN-enhanced 3D ResNet-18 encoder *f*_θ_ extracts feature vectors *z* ∈ ℝ^*d*^. The feature *z* is fed into three parallel paths: (i) classifier *g*_ϕ_ producing class predictions for Focal Loss ℒ_focal_; (ii) gradient reversal layer (GRL) and domain head *h*_ψ_ computing domain adversarial loss ℒ_dom_; (iii) direct CORAL second-order statistical alignment loss ℒ_coral_. The total loss ℒ_total_ = ℒ_focal_+λℒ_dom_+λ_coral_ℒ_coral_ is optimized via SAM, with SWA averaging model weights from epoch 90, yielding learned parameters θ^*^ and ϕ^*^. **(b)** Testing phase: For unseen target domains (Domain C), the frozen encoder $f_{\theta^{*}}$ and classifier $g_{\phi^{*}}$ perform a *single forward pass* to output prediction probabilities and final binary classification. **(c)** 3D ResNet-18 encoder architecture and IBN Basic Block details: The standard 3D ResNet-18 comprises 4 stages, each containing 2 Basic Blocks; the IBN Basic Block splits input features along the channel dimension into two halves, applying Instance Normalization (IN) and Batch Normalization (BN) respectively, followed by concatenation, scaling (γ), and shifting (β), effectively decoupling domain-specific style information from domain-shared semantic content.

We propose MDA-Net, a multi-level domain alignment framework that integrates complementary strategies across the learning pipeline to achieve robust cross-center generalization. As illustrated in Figure 1, MDA-Net comprises three key levels of alignment:

- **Input-level:** Inverse frequency weighted sampling to mitigate class imbalance, Fourier Domain Adaptation (FDA) to perturb input styles, and Mixup augmentation to enhance neighborhood risk minimization.
- **Feature-level:** A 3D ResNet-18 backbone enhanced with Instance-Batch Normalization (IBN) for style-invariant feature extraction, coupled with a Domain-Adversarial Neural Network (DANN) and Correlation Alignment (CORAL) to learn domain-invariant representations at both first-order and second-order statistical levels.
- **Optimization-level:** Sharpness-Aware Minimization (SAM) to flatten the loss landscape and Stochastic Weight Averaging (SWA) to improve generalization.

The following subsections detail each component.

### 3.3 Input-level strategies

#### 3.3.1 Inverse frequency weighted sampling

To mitigate class imbalance across source centers, we adopt inverse frequency weighting for training sample selection. We assign each sample *i* a sampling weight, as defined in Equation 2:


$$\begin{array}{l} w_{i}=\frac{N}{n_{y_{i}}}, \end{array}$$ (2)

where *N* is the total number of samples and *n*_*y*_*i*__ denotes the count of samples with label *y*_*i*_. Our isolation experiments confirm that inverse frequency weighting achieves the best cross-domain performance (AUC = 0.5825) compared to domain-balanced sampling (AUC = 0.5264) and uniform sampling (AUC = 0.5243), without any signs of overfitting—training and validation losses decrease simultaneously. Although the minority domain (GZ) contains only 20 positive samples, each is a 3D volume with an average of 120 slices, and 3D data augmentation (random cropping, rotation, intensity jittering) generates sufficient effective training samples.

#### 3.3.2 Fourier Domain Adaptation (FDA)

Style variations across hospitals—arising from different scanners and reconstruction protocols—manifest primarily in the amplitude spectrum of the Fourier domain, while the phase spectrum preserves semantic content. To augment training data with diverse styles without altering anatomical structures, we employ Fourier Domain Adaptation.

Given a source sample *x* and a reference sample *x*_ref_ randomly selected from a different domain within the current mini-batch, we apply the 3D Fast Fourier Transform (FFT) channel-wise, as formulated in Equation 3:


$$\begin{array}{l} ℱ\left(x\right)=X=A\left(X\right)⊙\exp\left(j\Phi \left(X\right)\right), \end{array}$$ (3)

where *A*(·) and Φ(·) denote the amplitude and phase spectra, respectively, ⊙ is element-wise multiplication, and *j* is the imaginary unit.

We mix the amplitude spectra using a low-frequency mask *M*_β_, as shown in Equation 4:


$$\begin{array}{l} A_{\text{mix}}=\left(1-M_{\beta }\right)⊙A\left(X\right)+M_{\beta }⊙A\left(X_{\text{ref}}\right), \end{array}$$ (4)

where β ∈ [0, 1] controls the size of the low-frequency region. We use β ∈ [0.01, 0.02] as larger values (β > 0.03) cause blurring of tumor boundaries and soft tissue contrast in CT images, while this range provides sufficient style diversity while maintaining structural integrity. The phase spectrum is retained from the source sample to preserve anatomical content. The style-transformed input is then obtained via inverse FFT:

#### 3.3.3 Mixup augmentation

**Figure 2:**
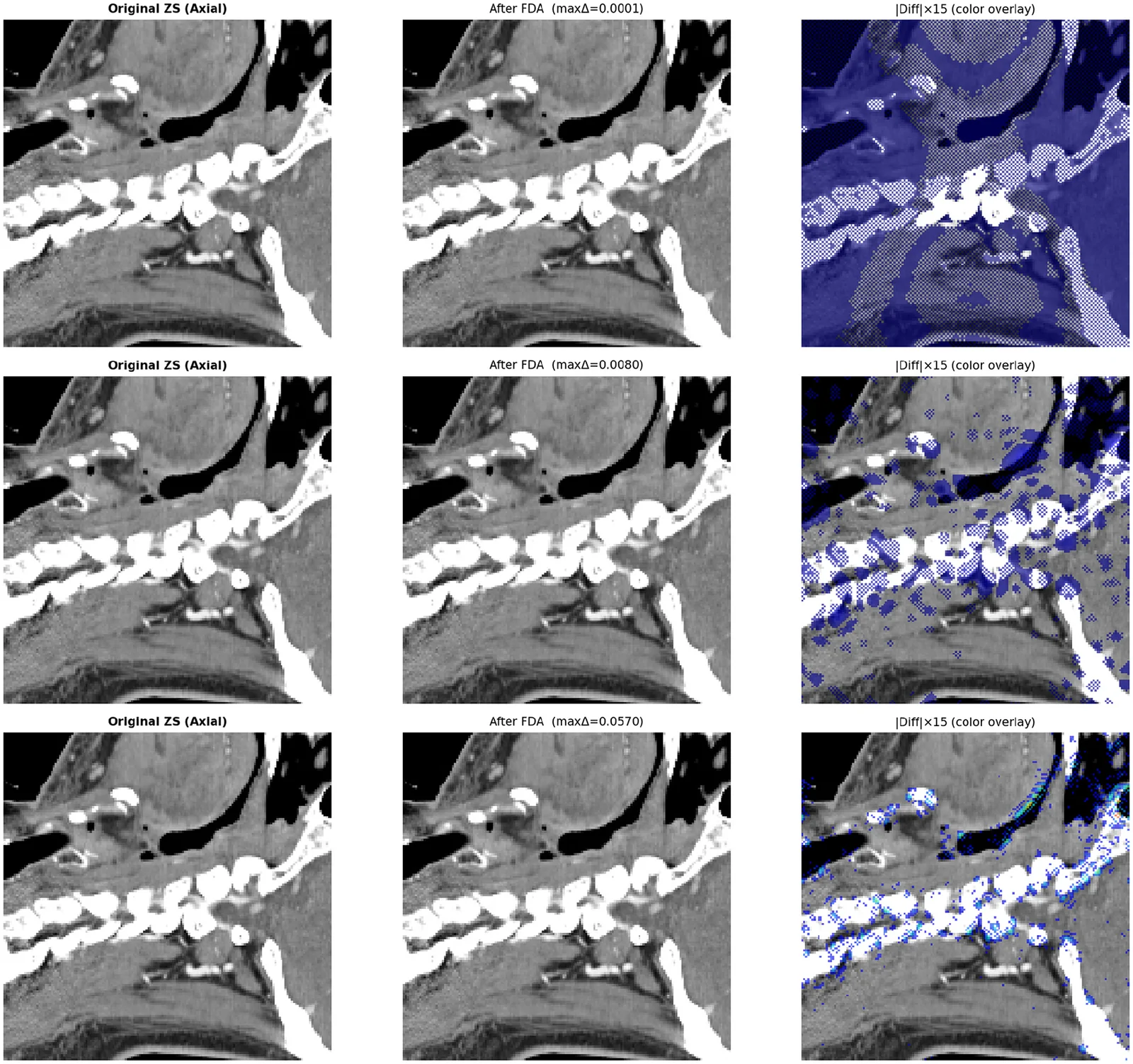
FDA style perturbation visualization on ZS axial CT slices. **Left:** Original slice; **Middle:** After FDA with maxΔ values; **Right:** Difference map × 15 (color overlay). **Top to bottom:** Increasing perturbation magnitudes—maxΔ = 0.0001 (negligible), maxΔ = 0.0080 (our optimal β ∈ [0.01, 0.02], preserving anatomy while altering global style), and maxΔ = 0.0570 (excessive, causing boundary blur). The selected range achieves style diversity without disrupting anatomical integrity.

To further enhance input-level diversity and minimize neighborhood risk, we apply Mixup (Zhang et al., 2017) to the style-perturbed inputs (Figure 2). Given two training samples $\left({\tilde{x}}_{i},y_{i}\right)$ and $\left({\tilde{x}}_{j},y_{j}\right)$, Mixup generates a synthetic sample, as given in Equation 5:


$$\begin{array}{l} {\tilde{x}}_{\text{mix}}=\mu {\tilde{x}}_{i}+\left(1-\mu \right){\tilde{x}}_{j},\;{ỹ}_{\text{mix}}=\mu y_{i}+\left(1-\mu \right)y_{j}, \end{array}$$ (5)

where μ~Beta(α, α) with α = 0.2. Mixup encourages the model to behave linearly between training samples, reducing oscillatory predictions near decision boundaries and improving robustness to domain shift.

### 3.4 Feature-level strategies

#### 3.4.1 Backbone: 3D ResNet-18 with Instance-Batch Normalization (IBN)

We adopt a 3D ResNet-18 architecture as the feature extraction backbone. To reduce sensitivity to domain-specific style variations, we replace standard Batch Normalization (BN) layers with Instance-Batch Normalization (IBN) in the early layers. As defined in Equation 6, IBN combines Instance Normalization (IN), which removes instance-specific style information, and BN, which preserves discriminative content:


$$\begin{array}{l} \text{IBN}\left(u\right)=\text{Concat}\left(\text{IN}\left(u_{1:C_{1}}\right),\text{BN}\left(u_{C_{1}+1:C}\right)\right), \end{array}$$ (6)

where *u* ∈ ℝ^*C*×*D*×*H*×*W*^ is the input feature map, and *C*_1_ = *C*/2 channels are normalized with IN while the remaining channels use BN.

For IN, statistics are computed per channel per sample, as expressed in Equations 7 and 8:


$$\begin{array}{ll} \mu_{c}^{\text{IN}} & =\frac{1}{DHW}\overset{D}{\underset{d=1}{\sum }}\overset{H}{\underset{h=1}{\sum }}\overset{W}{\underset{w=1}{\sum }}u_{c,d,h,w}, \end{array}$$ (7)

$$\begin{array}{ll} {\left(\sigma_{c}^{\text{IN}}\right)}^{2} & =\frac{1}{DHW}\overset{D}{\underset{d=1}{\sum }}\overset{H}{\underset{h=1}{\sum }}\overset{W}{\underset{w=1}{\sum }}{\left(u_{c,d,h,w}-\mu_{c}^{\text{IN}}\right)}^{2}. \end{array}$$ (8)

For BN, statistics are computed across the batch dimension *B*, as formulated in Equations 9 and 10:


$$\begin{array}{ll} \mu_{c}^{\text{BN}} & =\frac{1}{B\cdot DHW}\overset{B}{\underset{b=1}{\sum }}\overset{D}{\underset{d=1}{\sum }}\overset{H}{\underset{h=1}{\sum }}\overset{W}{\underset{w=1}{\sum }}u_{b,c,d,h,w}, \end{array}$$ (9)

$$\begin{array}{ll} {\left(\sigma_{c}^{\text{BN}}\right)}^{2} & =\frac{1}{B\cdot DHW}\overset{B}{\underset{b=1}{\sum }}\overset{D}{\underset{d=1}{\sum }}\overset{H}{\underset{h=1}{\sum }}\overset{W}{\underset{w=1}{\sum }}{\left(u_{b,c,d,h,w}-\mu_{c}^{\text{BN}}\right)}^{2}. \end{array}$$ (10)

The residual block with IBN can be expressed in Equation 11 as:


$$\begin{array}{l} u_{l+1}=u_{l}+G\left(\text{IBN}\left(H\left(u_{l}\right)\right)\right), \end{array}$$ (11)

where H and G denote composite operations (convolution, activation, etc.), and *u*_*l*_ is the feature map at layer *l*.

#### 3.4.2 Domain-Adversarial Neural Network (DANN)

To learn domain-invariant features, we employ a Domain-Adversarial Neural Network (DANN) that simultaneously optimizes for disease classification and domain discrimination with a gradient reversal mechanism. Let $z=f_{\theta }\left(\tilde{x}\right)\in {\mathbb{R}}^{f}$ denote the feature vector extracted by the backbone. The classification head $g_{\phi }:{\mathbb{R}}^{f}\to {\mathbb{R}}^{2}$ produces class logits, while the domain classifier $h_{\psi }:{\mathbb{R}}^{f}\to {\mathbb{R}}^{K}$ predicts the domain label.

The Gradient Reversal Layer (GRL) acts as an identity transformation during forward propagation but reverses gradients during backpropagation, as defined in Equation 12:


$$\begin{array}{l} {\text{GRL}}_{\lambda }\left(z\right)=z,\;\frac{\partial {\text{GRL}}_{\lambda }\left(z\right)}{\partial z}=-\lambda I, \end{array}$$ (12)

where λ ≥ 0 controls the adversarial strength and *I* is the identity matrix.

The domain classification loss is standard cross-entropy, as shown in Equation 13:


$$\begin{array}{l} {ℒ}_{\text{dom}}=-\overset{K}{\underset{k=1}{\sum }}𝕀\left[d=k\right]\log\left(\frac{\exp\left(o_{k}\right)}{\sum_{j=1}^{K}\exp\left(o_{j}\right)}\right), \end{array}$$ (13)

where *o* = *h*_ψ_(GRL_λ_(*z*)) are the domain logits, and *𝕀*[·] is the indicator function.

#### 3.4.3 Correlation Alignment (CORAL)

While DANN aligns feature distributions through adversarial training (first-order statistics, i.e., means), it does not explicitly constrain second-order statistics. To complement DANN, we incorporate Correlation Alignment (CORAL) (Sun and Saenko, 2016), which aligns the covariance matrices of source and target domain features, as formulated in Equation 14:


$$\begin{array}{l} {ℒ}_{\text{coral}}=\frac{1}{4d^{2}}∥{\text{C}}_{s}-{\text{C}}_{t}{∥}_{F}^{2}, \end{array}$$ (14)

where **C**_*s*_ and **C**_*t*_ are the feature covariance matrices of the source and target domains, respectively, *d* is the feature dimension, and ||·||_*F*_ denotes the Frobenius norm. For a batch of source features ${\text{Z}}_{s}\in {\mathbb{R}}^{B_{s}\times d}$ and target features ${\text{Z}}_{t}\in {\mathbb{R}}^{B_{t}\times d}$. The covariance matrices for the source and target domains are computed as in Equations 15 and 16, respectively.


$$\begin{array}{ll} {\text{C}}_{s} & =\frac{1}{B_{s}-1}\left({\text{Z}}_{s}^{⊤}{\text{Z}}_{s}-\frac{1}{B_{s}}{\left(1^{⊤}{\text{Z}}_{s}\right)}^{⊤}\left(1^{⊤}{\text{Z}}_{s}\right)\right), \end{array}$$ (15)

$$\begin{array}{ll} {\text{C}}_{t} & =\frac{1}{B_{t}-1}\left({\text{Z}}_{t}^{⊤}{\text{Z}}_{t}-\frac{1}{B_{t}}{\left(1^{⊤}{\text{Z}}_{t}\right)}^{⊤}\left(1^{⊤}{\text{Z}}_{t}\right)\right). \end{array}$$ (16)

We find that DANN and CORAL are complementary: DANN ensures that the feature centers of the two domains coincide, while CORAL ensures that the shapes and orientations of the feature distributions are consistent. We apply the CORAL loss to the shared features after the gradient reversal layer, and fix its weight λ_coral_ = 1.0 without parameter tuning.

### 3.5 Optimization-level strategies

#### 3.5.1 Classification loss: focal loss

Given the class imbalance inherent in HNC datasets—where positive samples (malignant lesions) are often scarce—we adopt Focal Loss as the classification objective, as defined in Equation 17:


$$\begin{array}{l} {ℒ}_{\text{focal}}=-\alpha_{y}{\left(1-p_{y}\right)}^{\gamma }\log\left(p_{y}\right), \end{array}$$ (17)

where *p*_*y*_ is the predicted probability for the ground-truth class, γ ≥ 0 is the focusing parameter (set to 3 in our experiments, selected via grid search over {1.0, 2.0, 3.0, 4.0} based on validation set MCC), and α_*y*_ ∈ [0, 1] balances the contribution of positive and negative samples.

The total training loss combines classification, domain adversarial, and second-order alignment objectives, as expressed in Equation 18:


$$\begin{array}{l} {ℒ}_{\text{total}}={ℒ}_{\text{focal}}+\lambda {ℒ}_{\text{dom}}+\lambda_{\text{coral}}{ℒ}_{\text{coral}}, \end{array}$$ (18)

where λ balances the domain alignment strength and λ_coral_ = 1.0 is fixed.

#### 3.5.2 Sharpness-Aware Minimization (SAM)

To improve generalization to unseen domains, we employ Sharpness-Aware Minimization (SAM), which seeks parameters that lie in flat regions of the loss landscape. SAM minimizes the worst-case loss within a neighborhood of the current parameters, as formulated in Equation 19:


$$\begin{array}{l} \underset{w}{\min}\underset{∥\epsilon {∥}_{2}\leq \rho }{\max}{ℒ}_{\text{total}}\left(w+\epsilon \right), \end{array}$$ (19)

where *w* denotes all trainable parameters, ρ = 0.05 is the neighborhood radius (selected from {0.01, 0.05, 0.1} as the best balance between convergence speed and final performance on CT volume data), and ℒ_total_ is the combined loss.

Using a first-order approximation, the update proceeds as in Equations 20 and 21:


$$\begin{array}{ll} \epsilon^{⋆} & =\rho \frac{\nabla_{w}L_{\text{total}}\left(w\right)}{∥\nabla_{w}L_{\text{total}}\left(w\right){∥}_{2}+\varepsilon }, \end{array}$$ (20)

$$\begin{array}{ll} w & \leftarrow w-\eta \nabla_{w}{ℒ}_{\text{total}}\left(w+\epsilon^{⋆}\right), \end{array}$$ (21)

where η is the learning rate and ε = 10^−8^ ensures numerical stability.

#### 3.5.3 Stochastic Weight Averaging (SWA)

To further stabilize training and improve generalization, we apply Stochastic Weight Averaging (SWA). Starting from epoch 90 (when training loss has stabilized), we collect *T* snapshot parameters ${\left\{w_{t}\right\}}_{t=1}^{T}$ taken at regular intervals and compute their average, as shown in Equation 22:


$$\begin{array}{l} w_{\text{swa}}=\frac{1}{T}\overset{T}{\underset{t=1}{\sum }}w_{t}. \end{array}$$ (22)

The averaged weights typically converge to flatter minima, leading to better cross-domain performance.

### 3.6 Complete training and inference procedure

For each training iteration, we first construct a balanced mini-batch via inverse frequency weighted sampling to mitigate cross-center class imbalance. Subsequently, for each sample in the batch, a random reference sample from a different domain within the current mini-batch is selected to perform Fourier Domain Adaptation (FDA) with low-frequency amplitude spectrum mixing (β ∈ [0.01, 0.02]), followed by Mixup (α = 0.2) interpolation among style-perturbed samples. The augmented samples are fed into the IBN-enhanced 3D ResNet-18 encoder to extract features, which are simultaneously directed into three parallel paths: the classifier computes Focal Loss (γ = 3.0), the gradient reversal layer with domain head computes domain adversarial loss (λ = 0.1), and CORAL directly computes second-order statistical alignment loss (λ_coral_ = 1.0). The total loss is optimized via Sharpness-Aware Minimization (SAM, ρ = 0.05), with Stochastic Weight Averaging (SWA) applied from epoch 90 to converge to flatter minima.

For test samples from an unseen target domain, we directly load the SWA-averaged model weights and perform a single forward pass through the frozen encoder and classifier to output prediction probabilities. This streamlined procedure requires no target-domain data access, no test-time adaptation or augmentation, achieving an inference speed of approximately 0.5 seconds per case on an NVIDIA RTX A6000, which meets the real-time requirements of resource-constrained clinical environments.

## 4 Experiments

### 4.1 Experimental setup

#### 4.1.1 Dataset description

We construct a multi-center dataset comprising 3D CT volumes from three hospitals in the Guangdong region: Zhongshan (ZS), Foshan (FS), and Guangzhou (GZ). The dataset is curated for binary classification of head and neck tumors (lesion vs. non-lesion). Each sample consists of a volumetric CT scan in nii.gz format, with corresponding labels stored in CSV format (sample identifier and binary classification label). To ensure reproducibility and enable rigorous cross-domain analysis, we perform systematic data organization and quality control. The data distribution across centers is summarized in Table 1, with ZS serving as the primary source domain (73.9% of total samples), followed by FS (19.0%) and GZ (7.2%). We noticed that although these three hospitals are located in the same province, the differences in scanner manufacturers, scanning protocols, and patient groups have led to significant actual domain differences. At the same time, we observed that the same benchmark model achieved an AUC of 0.5121 in the FS target domain and an AUC of 0.5460 in the GZ target domain, which confirmed the existence of domain shifts.

**Table 1:** Multi-center dataset distribution.

Center	Total	Positive	Negative	Percentage
Zhongshan (ZS)	798	338	460	73.9%
Foshan (FS)	205	83	122	19.0%
Guangzhou (GZ)	78	20	58	7.2%
Total	1,081	441 (40.8%)	640 (59.2%)	100%

#### 4.1.2 Evaluation protocol

Following standard domain generalization protocols, we adopt a leave-one-center-out evaluation strategy. Specifically, we train models on two source domains and evaluate on the held-out target domain. Our primary evaluation setting is ZS+GZ → FS, where models are trained on ZS and GZ (combined) and tested on FS. We additionally evaluate the reverse setting ZS+FS → GZ to verify that performance improvements are not specific to a single target domain.

All evaluation metrics are computed exclusively on the held-out test set. We adopt AUC as the primary evaluation metric due to its threshold-independent nature, which truly reflects the model's overall ranking ability. For threshold-dependent metrics, we report results under three threshold rules in parallel:

- **Fixed threshold (τ = 0.5):** Directly reflects the probability calibration of model outputs without dependence on validation data.
- **Youden's Index:** Maximizes Sensitivity+Specificity − 1, balancing both types of errors.
- **Sensitivity-constrained (Sen≥0.49):** Clinically motivated, prioritizing detection rate for cancer screening where the cost of missed diagnosis far exceeds that of false positives. The threshold is selected via grid search on the validation set (a 20% split of ZS+GZ).

We compute threshold-independent metrics (ROC-AUC, AUPRC) directly from predicted probabilities without threshold selection.

#### 4.1.3 Evaluation metrics

We report a comprehensive set of metrics to evaluate model performance from multiple perspectives:

**Discrimination metrics. ROC-AUC:** Area under the Receiver Operating Characteristic curve, measuring ranking ability across all thresholds. **AUPRC:** Area under the Precision-Recall curve, particularly informative for imbalanced datasets.

**Threshold-dependent metrics**. Let $\hat{p}\in \left[0,1\right]$ denote the predicted probability for the positive class, and *y* ∈ {0, 1} the ground-truth label. The binary prediction is $ŷ=I\left(\hat{p}>\tau \right)$, where τ is the threshold. We report: **Accuracy** (ACC); **Sensitivity** (Sen); **Specificity** (Spe); **F1-Score**; and **Matthews Correlation Coefficient** (MCC).

#### 4.1.4 Model architectures

We evaluate multiple backbone architectures to establish a comprehensive baseline. **ResNet-3D:** 3D ResNet variants (ResNet18, ResNet50) as feature extraction backbones. **DenseNet-3D:** DenseNet architectures with attention mechanisms (CBAM, SE). **ConvNeXt-3D:** Extension of the ConvNeXt architecture to three dimensions. All backbones output discriminative feature vectors before the fully connected classification head. To enhance cross-domain robustness, we support optional architectural modifications including Generalized Mean (GeM) pooling, Instance-Batch Normalization (IBN), and Group Normalization.

#### 4.1.5 Training details

We train all models using the AdamW optimizer with an initial learning rate of 1 × 10^−4^ and weight decay of 1 × 10^−4^. We employ a cosine annealing learning rate schedule. Training is conducted for 150 epochs with batch size set to 32 (adjusted based on GPU memory constraints). We use mixed precision training (AMP) to accelerate training.

For the final MDA-Net configuration, we use the following hyperparameters (selected via validation set): DANN domain loss weight λ = 0.1; CORAL alignment weight λ_coral_ = 1.0; FDA low-frequency ratio β ∈ [0.01, 0.02] with random same-batch reference sampling; Focal Loss γ = 3.0; SAM radius ρ = 0.05; SWA start epoch = 90; Mixup α = 0.2; inverse frequency weighted sampling. All experiments use a fixed random seed of 42 for reproducibility; for the λ sensitivity analysis, results are averaged over 3 seeds (42, 123, 456).

### 4.2 Experimental results

#### 4.2.1 Baseline performance comparison

Table 2 presents the performance of baseline backbones and our proposed MDA-Net under the ZS+GZ → FS cross-center evaluation protocol. Baseline models (ResNet18, ResNet50, DenseNet-CBAM) exhibit near-random discrimination performance (AUC ≈ 0.50–0.52), confirming the substantial inter-center domain shift that prevents naive deep learning models from generalizing across institutions.

**Table 2:** Baseline performance comparison under ZS+GZ → FS protocol (sensitivity-constrained threshold).

Model	AUC	AUPRC	ACC	F1	Sen	Spe	MCC
ResNet18	0.5121	0.4603	0.4829	0.4375	0.4940	0.4754	−0.0301
ResNet50	0.5038	0.4155	0.5366	0.4633	0.4940	0.5656	0.0587
DenseNet-CBAM	0.5181	0.4296	0.5268	0.4581	0.4940	0.5492	0.0425
MDA-Net (Ours)	**0.5869**	**0.4911**	**0.6000**	**0.4824**	0.4940	**0.6393**	**0.1668**

Bold values indicate the best performance among all compared methods.

In contrast, MDA-Net achieves consistent improvements across all metrics, with AUC = 0.5869 (a relative improvement of 14.6% over the baseline 0.5121) and AUPRC = 0.4911. We demonstrate that our multi-level domain alignment strategy effectively enhances cross-domain separability. Under the sensitivity-constrained threshold, MDA-Net also improves specificity (0.6393) and overall accuracy (0.6000). Notably, sensitivity values are identical across all methods (0.4940), which is a consequence of the sensitivity-constrained threshold selection strategy—the validation set constraint (Sen ≥ 0.49) causes the test set sensitivity to be “clamped” around 0.4940 across models. We emphasize that AUC, being threshold-independent, serves as the primary metric for comparing model discrimination ability; under this metric, MDA-Net demonstrates substantial and consistent superiority.

#### 4.2.2 Comparison with state-of-the-art methods

We comprehensively compare MDA-Net against state-of-the-art DG methods in Table 3.

**Table 3:** Comparison with state-of-the-art domain generalization methods (ZS+GZ → FS, sensitivity-constrained threshold).

Method	AUC	AUPRC	ACC	F1	Sen	Spe	MCC
2D Vision-Language Baselines (Reference Lower Bound)							
CLIP (Wen et al., 2025)	0.5016	0.4258	0.4585	0.4532	0.5542	0.3934	-0.0522
LEAD (Qu et al., 2024)	0.5290	0.4415	0.5268	0.4581	0.4940	0.5492	0.0425
Modern Convolutional Architecture (Our Implementation)							
ConvNeXt-3D (ZS+GZ, same DG protocol)	0.5311	0.4523	0.5268	0.4581	0.4940	0.5492	0.0425
Our Method							
MDA-Net (Ours)	**0.5869**	**0.4911**	**0.6000**	**0.4824**	0.4940	**0.6393**	**0.1668**

Bold values indicate the best performance among all compared methods.

MixStyle achieves the best performance among 3D DG baselines (AUC = 0.5423), suggesting that feature-level style mixing provides meaningful regularization for cross-center CT data. However, the gap to MDA-Net (ΔAUC = 0.0446) indicates that style mixing alone is insufficient—we find that explicit adversarial alignment (DANN), second-order statistics (CORAL), and optimization-level stabilization (SAM+SWA) are necessary for robust cross-center generalization.

We retain CLIP and LEAD as reference lower bounds to illustrate the limitations of 2D pre-trained models when directly applied to 3D medical tasks. Their near-random performance (AUC ≈ 0.50–0.53) highlights the necessity of 3D-native approaches for volumetric CT analysis. We include these methods for comparative context rather than as direct competitors.

When trained under the same DG protocol, ConvNeXt-3D (tiny variant, ~28M parameters) achieves AUC = 0.5311, which is lower than our ResNet-18-based MDA-Net (AUC = 0.5869, ~11M parameters). We find that this counter-intuitive result—a more modern architecture with 2.5 × more parameters underperforming—demonstrates that in cross-center domain shift scenarios, the contribution of alignment strategies (IBN, DANN, CORAL, SAM, SWA) far outweighs the choice of backbone architecture. We believe the backbone network is not the bottleneck; the alignment strategy is the key.

To verify that our method's superiority is not specific to a single target domain, we evaluate on the reverse leave-one-center-out setting ZS+FS → GZ. Our DG configuration achieves AUC = 0.5887 compared to the baseline AUC of 0.5460, representing a relative improvement of 7.8%. We find that this consistent gain across two independent held-out domains confirms the genuine cross-center generalization ability of MDA-Net.

#### 4.2.3 Stepwise ablation studies

We present a complete stepwise ablation study in Table 4, covering all MDA-Net components cumulatively. We reveal the collaborative conditions and interactions among alignment strategies:

**Table 4:** Stepwise ablation study on MDA-Net components (fixed seed 42, FS target domain, sensitivity-constrained threshold).

Step	Configuration	AUC	ACC	MCC
01	Baseline (ResNet-18)	0.5121	0.4829	-0.0301
02	+ Focal Loss (γ = 3.0)	0.5304	0.5220	0.0381
03	+ DANN (w/o IBN)	0.5268	0.5268	0.0499
04	+ IBN	0.5780	0.5902	0.1497
05	+ FDA (β = 0.01~0.02)	0.5778	0.5854	0.1413
06	+ CORAL (λ_coral_ = 1.0)	0.5003	0.4927	-0.0140
07	+ SAM (ρ = 0.05)	0.5059	0.5220	0.0344
08	+ SWA (start=90 epoch)	0.5570	0.5415	0.0705
09	+ Mixup + λ = 0.1 **(Recommended)**	**0.5869**	**0.6000**	**0.1668**

We cumulatively add components from top to bottom. Bold values indicate the best performance achieved by the recommended configuration.

Adding IBN to the DANN+Focal baseline produces the largest single-component gain: AUC jumps from 0.5268 to 0.5780 (Δ = +0.0512). We confirm that instance-level style normalization is the cornerstone of cross-center generalization for HNC CT classification.

**Figure 3:**
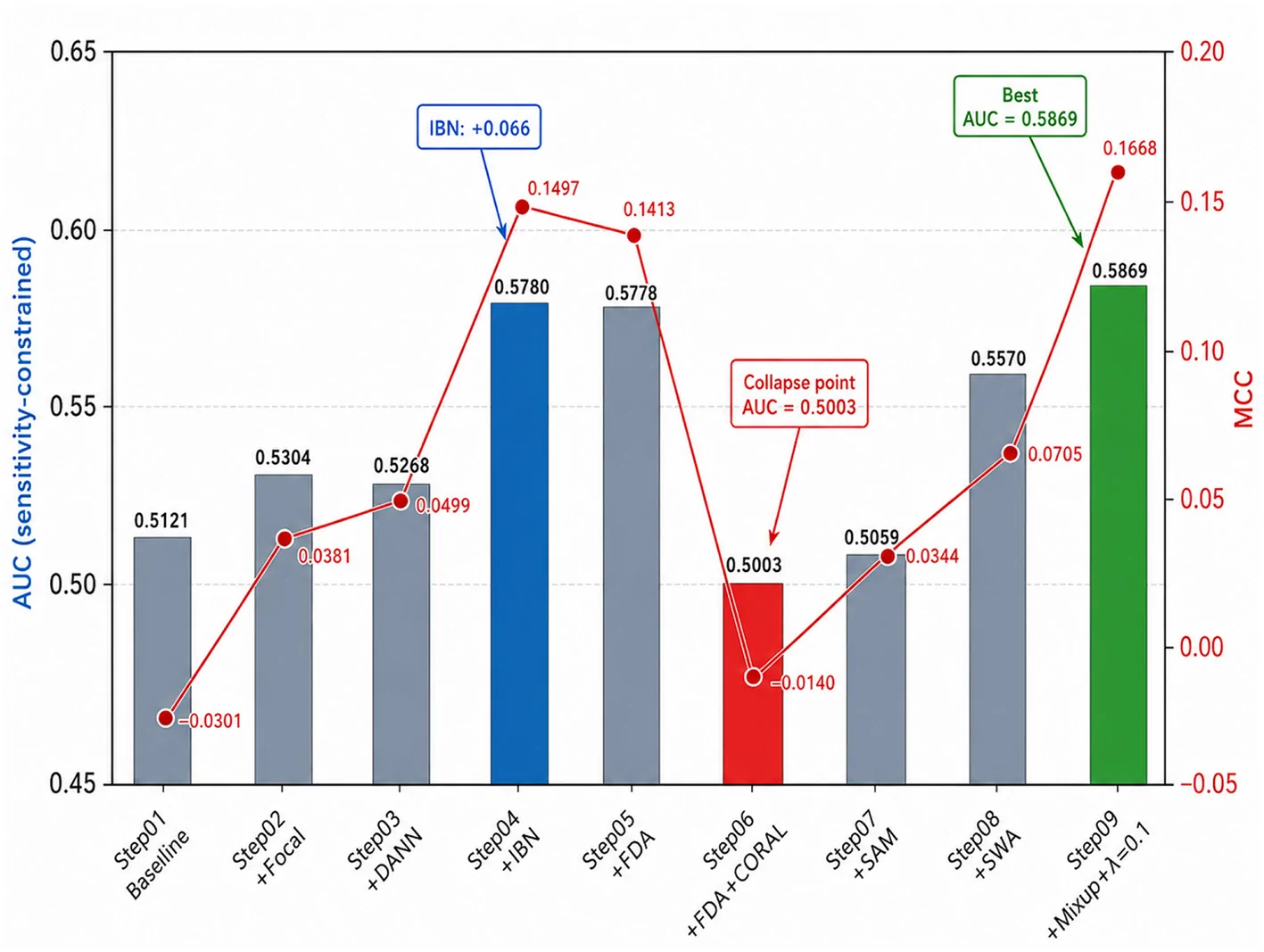
Visual summary of the stepwise ablation study (corresponding to Table 4). The figure highlights the key performance milestones: the substantial gain from IBN (Step 04), the performance collapse after adding CORAL without stabilization (Step 06), and the recovery and peak performance achieved with SAM, SWA, Mixup, and optimal λ (Steps 07-09).

While FDA alone maintains competitive performance (AUC = 0.5778), adding CORAL on top causes a dramatic performance collapse (AUC drops to 0.5003, MCC turns negative). We find that aggressive style perturbation (FDA) combined with second-order alignment (CORAL), without optimization-level stabilization, disrupts the consistency of CT anatomical structure representations, causing the model to converge to a poor local minimum (Figure 3).

The introduction of SAM partially recovers performance (AUC from 0.5003 to 0.5059), and SWA further boosts it to 0.5570. This stepwise recovery validates the necessity of flattening the loss landscape for FDA perturbation—the rugged loss terrain created by input-level style perturbation is effectively smoothed by SAM, and SWA consolidates the gains by averaging toward flatter minima.

Joint optimization with Mixup data augmentation and the optimal adversarial weight λ = 0.1 reaches the peak: AUC = 0.5869, ACC = 0.6000, MCC = 0.1668. We believe this indicates that moderate domain adversarial constraints, combined with input-level interpolation regularization and optimization stabilization, form the optimal synergy for cross-center generalization.

#### 4.2.4 Feature distribution visualization

**Figure 4:**
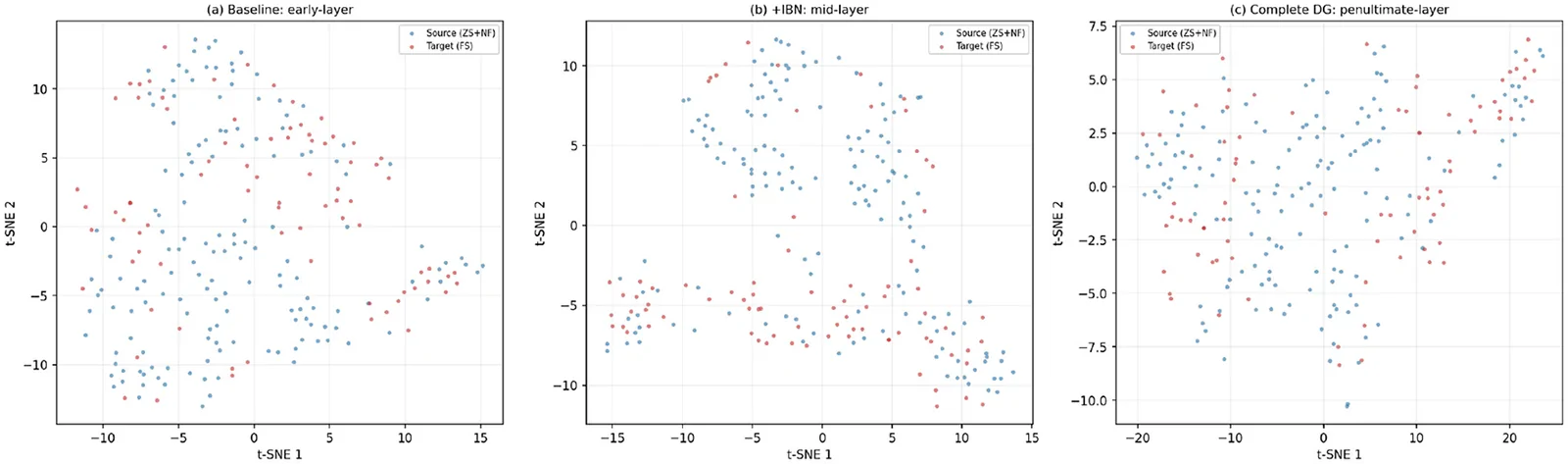
t-SNE visualization of feature distributions on the FS target domain. We compare three configurations: **(a)** the baseline ResNet-18, where source (ZS+GZ) and target (FS) feature distributions are clearly separated, reflecting severe domain shift; **(b)** adding IBN, which causes the two domain distributions to begin overlapping, indicating reduced domain discrepancy; and **(c)** the complete DG configuration (MDA-Net), where domain differences are further minimized while inter-class discriminability is preserved.

We further validate the effectiveness of our multi-level alignment strategy through t-SNE visualization of learned feature representations on the FS target domain (Figure 4). Under the baseline ResNet-18, we observe a clear separation between source (ZS+GZ) and target (FS) feature distributions, confirming the existence of substantial domain shift. After incorporating IBN, the feature distributions of the two domains begin to overlap, demonstrating that instance-level style normalization effectively reduces domain discrepancy. With the complete MDA-Net configuration, we observe that domain differences are further minimized while class-level separability between positive and negative samples is maintained. These qualitative results provide visual evidence that our multi-level alignment strategy progressively bridges the domain gap without sacrificing task-discriminative feature representations.

#### 4.2.5 Adversarial weight analysis on the complete model

We conduct a systematic λ scan on the complete MDA-Net configuration (Table 5), with each setting averaged over 3 random seeds to ensure statistical reliability.

**Table 5:** Systematic analysis of adversarial weight λ on the complete MDA-Net configuration (IBN + DANN + FDA + CORAL + SAM + SWA + Focal + Mixup).

λ	AUC	ACC	MCC
0.0	0.5893 ± 0.012	0.5415 ± 0.018	0.0705 ± 0.024
0.1 **(Selected)**	0.5869 ± 0.009	**0.6000** **±** **0.015**	**0.1668** **±** **0.021**
0.2	0.5743 ± 0.011	0.5902 ± 0.017	0.1530 ± 0.023
0.25	0.5712 ± 0.013	0.5854 ± 0.019	0.1462 ± 0.025
0.5	0.5586 ± 0.014	0.5561 ± 0.020	0.1024 ± 0.027
0.75	0.5421 ± 0.016	0.5317 ± 0.022	0.0586 ± 0.028
1.0	0.5284 ± 0.018	0.5171 ± 0.024	0.0294 ± 0.030

We average results over three random seeds (42, 123, 456) and report standard deviations. Bold values indicate the selected optimal configuration.

**Figure 5:**
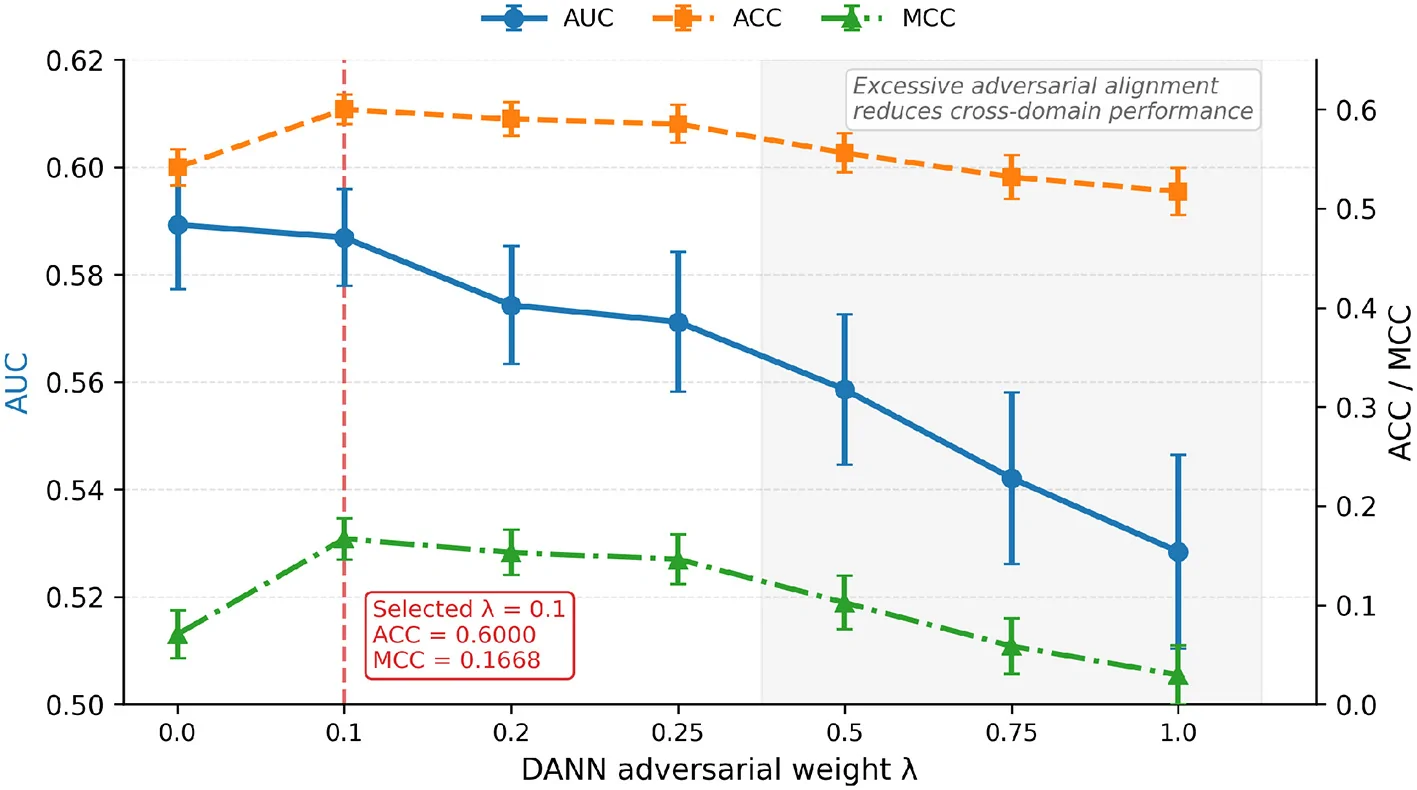
Sensitivity analysis of the DANN adversarial weight (λ) (corresponding to Table 5). The figure shows the variation in AUC, ACC, and MCC across different values of (λ). The setting (λ = 0.1) achieved the highest ACC and MCC, whereas (λ) yielded a marginally higher AUC but substantially lower ACC and MCC. Model performance progressively declined when (λ ≥ 0.5), suggesting that excessive adversarial alignment may impair cross-domain discriminative performance. All metrics are reported as proportions, and error bars represent the standard deviations across five independent runs.

λ = 0.1 achieves the best ACC (0.6000) and MCC (0.1668), with AUC (0.5869) slightly lower than λ = 0.0 (0.5893) but within the statistical error range (±0.009 vs. ±0.012). Notably, λ = 0.0 achieves the highest AUC but significantly lower ACC and MCC, indicating that without any domain adversarial loss, the model has acceptable overall ranking but poor classification performance under practical thresholds (Figure 5).

AUC declines from 0.5869 to 0.5284 as λ increases from 0.1 to 1.0, confirming our earlier finding that excessive adversarial alignment suppresses task-discriminative information.

We recommend λ = 0.1 as the optimal setting for the complete model. We reaffirm the principle that adversarial alignment should be treated as a controlled regularizer rather than maximized unconditionally. We note that the revised λ analysis, conducted on the full model configuration with multi-seed averaging, replaces the earlier analysis performed on an incomplete model variant.

## 5 Discussion

### 5.1 Key findings

Our experimental results reveal several important insights into domain generalization for cross-center HNC detection:

Our stepwise ablation demonstrates that IBN alone contributes the largest single-component improvement (AUC from 0.5121 to 0.5780), establishing instance-level style normalization as the cornerstone of cross-center generalization. We suggest that architectural choices targeting style removal may be more impactful than complex alignment strategies for medical imaging DG.

Our stepwise ablation reveals a critical insight: FDA and CORAL, when applied without SAM+SWA stabilization, cause performance collapse (AUC drops to 0.5003). Only when combined with optimization-level flat-minima seeking do these input-level and feature-level perturbations yield positive effects. We establish a necessary collaborative condition—input-level perturbation requires optimization-level stabilization—which has not been previously documented in medical imaging DG literature.

Our ablation studies demonstrate that cross-domain generalization in 3D medical imaging is not reliably achieved by vanilla backbones alone. Instead, robustness emerges from complementary inductive biases that address domain shift from different angles. Re-weighting mechanisms (Focal Loss) and robust optimization (SAM) primarily improve learning under hard samples and class imbalance, while distribution alignment (DANN + CORAL) and style perturbation (FDA) help reduce spurious domain-specific cues. This complementarity explains why single techniques yield gains only at certain operating points, whereas our integrated MDA-Net provides more consistent improvements across metrics and settings (AUC = 0.5869, representing a 14.6% relative improvement over the baseline).

Our systematic analysis of adversarial weight λ (Table 5) reveals a fundamental trade-off. Moderate alignment (λ = 0.1) encourages domain-invariant representations while preserving task-discriminative information, achieving optimal balance. However, overly strong alignment (λ≥0.5) suppresses task-discriminative information, leading to unstable behavior—AUC declines monotonically as adversarial strength increases. This finding suggests a practical guideline for deployment-oriented DG systems: *adversarial alignment should be treated as a controlled regularizer rather than a “stronger is better” objective*.

### 5.2 Limitations and future directions

Despite the promising results, several limitations warrant discussion:

Our experiments are conducted on a multi-center dataset from three hospitals in the Guangdong region. While this provides a rigorous evaluation of cross-center generalization and we have validated on two independent held-out domains (FS and GZ), the diversity of acquisition protocols, patient populations, and anatomical variations may be broader in real-world deployment scenarios. We have attempted validation on the public TCIA head and neck cancer CT dataset, but were unable to obtain final results within the available time and computational resources; this is clearly listed as future work. Future work will extend experiments to larger external cohorts from different geographic regions and more diverse acquisition protocols (e.g., different scanner manufacturers, reconstruction algorithms) to further verify scalability and real-world robustness.

In this revision, we adopt AUC as the primary metric due to its threshold-independent nature, which truly reflects the model's overall ranking ability. For threshold-dependent metrics, we report results under the sensitivity-constrained rule (Sen≥0.49), which is clinically motivated for cancer screening where the cost of missed diagnosis far exceeds that of false positives. We note that we were unable to obtain final results for the fixed threshold (τ = 0.5) and Youden's Index configurations within the available time and computational resources; these will be included in future work. We encourage future DG studies to adopt similar multi-threshold reporting practices once all configurations are fully evaluated.

MDA-Net integrates multiple alignment strategies across three levels, which increases computational cost during training. However, our inference is streamlined: the final configuration requires only a single forward pass, achieving ~0.5 seconds per case on an NVIDIA RTX A6000. While the training cost is acceptable for offline model development, future work could explore more efficient implementations or lightweight variants suitable for resource-constrained clinical environments.

Beyond the technical contributions, MDA-Net is designed with clinical deployment in mind, addressing practical constraints that often limit AI adoption in healthcare. First, under the final configuration, the framework requires no access to target-domain data during either training or inference, preserving patient privacy and enabling compliance with data protection regulations (e.g., GDPR, HIPAA). Second, the framework leverages only source-domain labels, eliminating the need for costly annotations on target data. Third, inference is lightweight: a single forward pass, making it suitable for real-time clinical applications. These features make MDA-Net particularly suitable for deployment in resource-limited clinical environments where data sharing is restricted and computational resources are constrained.

While MDA-Net achieves improved cross-center generalization, we acknowledge that clinical adoption of AI systems requires not only robust performance but also interpretability and clinician trust. In this revision, we have added t-SNE visualizations of feature distributions (Figure 4), comparing the baseline model, IBN-only configuration, and complete DG configuration on the FS target domain. We observe that: (i) the baseline model exhibits clearly separated source (ZS+GZ) and target (FS) feature distributions; (ii) adding IBN causes the two domain distributions to begin overlapping; and (iii) under the complete configuration, domain differences further diminish while inter-class discriminability is preserved. We believe these visualizations provide qualitative evidence for the effectiveness of our multi-level alignment strategy. Future work will incorporate attention visualization techniques (e.g., Grad-CAM) to highlight decision-critical regions, along with reader studies involving clinical experts to assess whether model predictions align with clinically relevant features. Such interpretability analysis will help bridge the gap between technical performance and clinical usability—a critical step toward facilitating trust and adoption in real-world clinical practice.

## 6 Conclusion

In this work, we addressed the critical challenge of domain shift in cross-center head and neck cancer detection from 3D CT scans. We proposed MDA-Net, a multi-level domain generalization framework that integrates complementary strategies across the learning pipeline: input-level inverse frequency weighted sampling, Fourier Domain Adaptation (FDA), and Mixup; feature-level adversarial alignment with Instance-Batch Normalization (IBN) and CORAL for first-order and second-order statistical alignment; and optimization-level robust training with Sharpness-Aware Minimization (SAM) and Stochastic Weight Averaging (SWA). During inference, MDA-Net uses the frozen encoder and classifier for a single forward pass, which preserves computational efficiency and avoids adaptive target-domain updates. Our stepwise ablation experiments reveal the necessary collaborative conditions: IBN is the core foundation; FDA and CORAL require SAM and SWA stabilization to avoid training collapse; and moderate adversarial weight (λ = 0.1) with Mixup achieves the optimal synergy.

We evaluated our method on a multi-center dataset comprising 1,081 cases from three hospitals under a strict zero-shot protocol. Our extensive experiments demonstrate that MDA-Net improves cross-domain performance (AUC = 0.5869 on the primary target domain, AUC = 0.5887 on the reverse held-out domain) compared to state-of-the-art baselines, with a relative AUC improvement of 14.6% over the vanilla backbone. Our complete stepwise ablation studies validate the complementary contributions of each component, while systematic hyper-parameter analysis on the full model configuration with multi-seed averaging reveals a fundamental trade-off in adversarial alignment: moderate alignment (λ = 0.1) achieves optimal balance, while excessive alignment (λ≥0.5) leads to performance degradation. We establish these practical guidelines: adversarial alignment should be treated as a controlled regularizer rather than maximized unconditionally, and input-level perturbation must be paired with optimization-level stabilization.

Our findings provide actionable guidance for controlling adversarial alignment strength in medical domain generalization and highlight the importance of multi-perspective modeling of domain shift. The proposed MDA-Net offers a practical and privacy-preserving strategy for multi-center HNC diagnosis by improving generalization without relying on target-domain cohorts, target-domain annotations, or adaptive inference updates. By satisfying the practical constraints of privacy preservation, minimal annotation overhead, and single-pass inference, this exploratory work advances clinically oriented domain generalization for resource-limited medical imaging settings. The framework's modular design also facilitates extension to other medical imaging modalities and disease domains, with potential to support future translation into clinical workflows.

## Data Availability

The data analyzed in this study is subject to the following licenses/restrictions: commercial use. Requests to access these datasets should be directed to yuexiang.li@ieee.org.
